# The Acquisition of Target Dependence by Developing Rat Retinal Ganglion Cells

**DOI:** 10.1523/ENEURO.0044-14.2015

**Published:** 2015-07-10

**Authors:** Colette Moses, Lachlan P.G. Wheeler, Chrisna J. LeVaillant, Anne Kramer, Marisa Ryan, Greg S. Cozens, Anil Sharma, Margaret A. Pollett, Jennifer Rodger, Alan R. Harvey

**Affiliations:** 1 School of Anatomy, Physiology, and Human Biology, The University of Western Australia, Perth, Western Australia 6009, Australia; 2 School of Animal Biology, The University of Western Australia, Perth, Western Australia 6009, Australia; 3 Department of Neuroscience, Karolinska Institutet, 17177 Stockholm, Sweden; 4 Western Australian Neuroscience Research Institute, Nedlands, Western Australia 6009, Australia

**Keywords:** BDNF, neurotrophic factors, programmed cell death, retinal ganglion cells, target dependence

## Abstract

Similar to neurons in the peripheral nervous system, immature CNS-derived RGCs become dependent on target-derived neurotrophic support as their axons reach termination sites in the brain. To study the factors that influence this developmental transition we took advantage of the fact that rat RGCs are born, and target innervation occurs, over a protracted period of time. Early-born RGCs have axons in the SC by birth (P0), whereas axons from late-born RGCs do not innervate the SC until P4-P5. Birth dating RGCs using EdU allowed us to identify RGCs (1) with axons still growing toward targets, (2) transitioning to target dependence, and (3) entirely dependent on target-derived support. Using laser-capture microdissection we isolated ∼34,000 EdU^+^ RGCs and analyzed transcript expression by custom qPCR array. Statistical analyses revealed a difference in gene expression profiles in actively growing RGCs compared with target-dependent RGCs, as well as in transitional versus target-dependent RGCs. Prior to innervation RGCs expressed high levels of BDNF and CNTFR α but lower levels of neurexin 1 mRNA. Analysis also revealed greater expression of transcripts for signaling molecules such as MAPK, Akt, CREB, and STAT. In a supporting *in vitro* study, purified birth-dated P1 RGCs were cultured for 24-48 h with or without BDNF; lack of BDNF resulted in significant loss of early-born but not late-born RGCs. In summary, we identified several important changes in RGC signaling that may form the basis for the switch from target independence to dependence.

## Significance Statement

During brain development many neurons die around the time neural connections are established. This cell loss is thought to be from competition between neurons for limited amounts of target-derived trophic support; responsive neurons receiving sufficient amounts of such factors survive. But what factors sustain developing neurons prior to target innervation? We took advantage of the fact that rat RGCs are born, and target innervation occurs, over a protracted time period. Using laser-capture microdissection of birth-dated RGCs we compared gene expression in neurons prior to, during, and after innervation of central targets. We identified numerous changes in RGC signaling that may form the basis for the switch from target independence to dependence and from axonal elongation to arborization/synaptogenesis.

## Introduction

Programmed cell death (PCD) occurs throughout the developing nervous system and is a crucial step in the maturation of neural circuitry. For example, in the developing rat visual system PCD results in a loss of at least 50% of the maturing RGC population (Dreher et al., 1983; Perry et al., 1983; Crespo et al., 1985; Harvey and Robertson, 1992). A key element in regulating the onset and distribution of PCD is the availability of neurotrophic factor support. In both the CNS and PNS, removal or addition of neurotrophic factors, respectively, increases or decreases the amount of PCD (PNS: Levi-Montalcini and Angeletti, 1968; Thoenen and Barde, 1980; Hamburger and Yip, 1984; Oppenheim 1991; CNS: Cui and Harvey, 1995; Cohen-Cory et al., 1996; Ma et al., 1998; Spalding et al., 1998, 2005). Such findings are consistent with the neurotrophic hypothesis (Davies, 1996), which proposes that neurons with axons in target sites compete for limited amounts of target-derived neurotrophic factors, and only responsive neurons that receive sufficient amounts of these factors survive.

Developing neurons with axons en route to targets survive independent of support from target-derived trophic factors. However, studies on cranial sensory neurons and lower motoneurons (Davies et al., 1987; Vogel and Davies, 1991; Mettling et al., 1995; Enokido et al., 1999) suggest that there is a maturational switch to dependency on target-derived factors as axons grow into such targets. Additionally in the PNS, the further axons have to grow the longer it takes for their parent neurons to switch on their trophic dependency (Davies, 1989; Vogel and Davies, 1991). Importantly, in PNS neurons the transition to target dependency is delayed by the same amount of time *in vitro* as it would be *in vivo* (Davies et al., 1987; Davies, 1989; Vogel and Davies, 1991), suggesting it is an intrinsic property of those neurons.

A similar phenomenon may occur in the visual system. In chick RGCs, a switch from target independence to dependence has been reported (Rodriguez-Tébar et al., 1989). In rat, RGCs first appear at ∼E13 and genesis continues until E19/E20 (Reese and Colello, 1992; Rapaport et al., 2004). Soon after differentiation, RGCs begin to extend axons (Waid and McLoon, 1995), almost all of which project to the contralateral SC (Sefton et al., 2004). Axons of first-born RGCs reach the SC at ∼E16.5 (Lund and Bunt 1976; Bunt et al., 1983), a delay of 2-3 d, whereas axons of RGCs born on E18/E19 do not reach the SC until 4-6 d after birth, a delay of 8-9 d (Dallimore et al., 2002). Progressive innervation of the brain by different populations of RGCs as they mature has also recently been described in the mouse (Osterhout et al., 2014). In the rat, early-born RGCs begin PCD before birth whereas late-born RGCs only die between P4 and P6 (Dallimore et al., 2010), thus a switch to target dependency should be revealed by differences in the time course of PCD in early-born versus late-born RGCs.

Previous studies have documented changes in gene expression in developing RGCs defined solely on the basis of the age of the animal from which the cells were obtained ( Trimarchi et al., 2007; Wang et al., 2007; Moore et al., 2009). However, because of the prolonged neurogenesis of RGCs in rats, at any given prenatal or postnatal age RGCs are at different stages of maturation, constituting a heterogeneous population (Dallimore et al., 2002, 2010). Thus to more definitively characterize the factors that contribute to the switch to target dependency in maturing RGCs, at different times after birth we analyzed and compared gene expression in neonatal rat RGCs that had been identified and selected purely on the basis of their day of neurogenesis.

RGCs born on E15 or E18 were labeled with pulses of EdU. RGCs were then investigated at different stages of target ingrowth: before axon ingrowth (E18-labeled pups killed at P0 or P1), during ingrowth (E18-labeled pups at P5), and after innervation (E15-labeled pups at P0). EdU-positive (^+^) RGCs were isolated from cryosections using single-cell laser-capture microdissection (LCM) and RNA extracted for qPCR. We selected genes that are either involved in trophic factor signaling or have been implicated in RGC survival and/or axonal outgrowth. Pathway-specific discriminant analysis was used to compare gene expression profiles between RGCs with axons already in the SC and RGCs with axons still growing toward the SC and other major central target sites. Because the data from the LCM studies suggested changes in BDNF signaling in maturing RGCs, we also compared *in vitro* the trophic requirements of purified P1 RGCs labeled on either E15 or E18 with BrdU.

## Materials and Methods

### Animals

Time-mated female Wistar rats (*n* = 10) were used for EdU injections and gene expression studies. EdU was used for the *in vivo* qPCR experiments because the protocols for visualizing BrdU result in RNA degradation, and an earlier pilot study found that it was not possible to capture BrdU-labeled cells with LCM while maintaining RNA integrity. E15 or E18 (day after mating = E0) pregnant rats were anesthetized with isoflurane (4% induction and 2% maintenance in 20% O_2_/80% N_2_O) and injected with EdU (20 mg/kg maternal body weight, i.p.) two times during the day (at 10 A.M. and 3 P.M.) to ensure prolonged bioavailability (Zeng et al., 2010). Procedures were approved by institutional (UWA Animal Ethics Committee) and government (NHMRC) guidelines.

### Tissue collection and processing

Parturition occurred on E22/22.5 (day of birth = P0). P0, P1, or P5 pups were deeply anesthetized with 0.2 ml intraperitoneal pentobarbital sodium (Lethabarb; Virbac) and perfused transcardially with 4% paraformaldehyde in diethylpyrocarbonate-treated 0.1 m PBS (dPBS). Eyes were harvested immediately after fixation and the cornea and lens removed in dPBS, leaving the eyecup with retina attached. Retinas were postfixed in 4% paraformaldehyde for 1 h, immersed in 30% sucrose in dPBS for 1 h, and then into increasing concentrations of Jung Tissue Freezing Medium (Leica Microsystems; 25, 50, 75, and 100%; 1 h immersion at each stage at 4°C) before being frozen. Eyecups were cryosectioned at 10 μm and every second section mounted onto Menzel***–***Gläser SuperFrost Plus glass slides (usually eight retinal sections per slide), ensuring that the eyecup was positioned so that the central to peripheral retina was represented in every section. Instruments and cryostat surfaces were treated with RNaseZap (Ambion) cleaning solution and 70% ethanol before use. Sections were stored at −80°C prior to immunohistochemistry and laser capture (see below).

Multiple retinas were allocated to individual groups, each group constituting a biological replicate that gave a distinct sample of microdissected RGCs for mRNA analysis. mRNA analysis experiments were performed in two separate rounds. For an initial pilot LCM experiment we had estimated that 800 RGCs would yield ∼8 ng of total RNA (10 pg per neuron); however, when many genes failed to amplify during qPCR it became clear that we had insufficient RNA of necessary quality to perform the desired analysis; thus, a second more extensive experiment was performed to capture a significantly greater number of EdU^+^ RGCs. The initial pilot experiment compared five replicate groups of E18 EdU RGCs with five replicate groups of E15 EdU RGCs, both at P0 (E18/P0 and E15/P0 label/killed, respectively). Five retinas, randomly selected from different litters, were pooled to form each replicate group (except for two E15 groups, one of which had three retinas and one had four). The second round of laser capture compared E18 RGCs at P1 (E18/P1, five replicate groups) with E18 at P5 (E18/P5, five replicate groups) and E15 at P0 (E15/P0, three replicate groups). Each of these groups contained RGCs from five different rat pups. For textual clarity, the E15/P0 RGCs now will be described as “target-dependent RGCs,” E18/P0 or P1 RGCs as “growing RGCs,” and E18/P5 RGCs as “transitional RGCs.”

### EdU and Brn3a immunohistochemistry

EdU detection was performed using the Click-iT EdU Alexa Fluor 488 Imaging Kit (Invitrogen). Sections were permeabilized with 0.2% Triton X-100 (Progen Industries) in dPBS for 30 min, washed with 3% protease-free BSA (Sigma-Aldrich) in dPBS (2 × 5 min), then incubated with 300 μl reaction mixture prepared according to kit directions for 30 min. In the first pilot run, cells were not double labeled with Brn3a to distinguish RGCs, because at P0 there are as yet no displaced amacrine cells in the GCL (Perry, 1981). In the more extensive follow-up experiment we also wished to obtain laser-captured E18 RGCs from P5 retinas at a time when their axons are in the process of innervating the SC; however, at this age potentially EdU^+^ amacrine cells are now present in the GCL. Thus for all groups in this second series we additionally immunostained retinal sections for Brn3a protein, an established marker for RGCs projecting to the SC (Nadal-Nicolás et al., 2009, 2012). Retinal sections were washed with dPBS (2 × 10 min) and incubated overnight at 4°C with anti-Brn3a goat primary antibody (AB; Santa Cruz Biotechnology, SC-31984) 1:100 in antibody diluent (10% normal horse serum and 0.2% Triton X-100 in dPBS). After washes, sections were incubated in donkey anti-goat Cy3 (Jackson ImmunoResearch, 705-166-147; 1:200 in antibody diluent) for 2 h.

### LCM

For both the pilot and second qPCR experiments, LCM was undertaken using a Carl Zeiss PALM CombiSystem. Retinal sections were viewed at 20× magnification; EdU^+^ cells (first experiment) or EdU^+^/Brn3a^+^ RGCs (second experiment) were tagged using PALM Robo software and catapulted into a 0.5 ml PCR tube cap containing 40 μl proteinase K digest buffer (Qiagen). Only those cells that were heavily and evenly labeled for EdU (first experiment) or both EdU and Brn3a (second experiment) were selected. Focus and energy settings were adjusted for each new slide prior to loading the collection cap to ensure a single cell was catapulted with each shot. After catapulting, sections were again checked to ensure single cells had been captured. Approximately 200 cells were dissected into each cap over a period of 1 h before the cap was transferred to −80°C. In the initial experiments ∼800 cells were collected for each of the five E18 replicate groups, and ∼1000-1200 cells for each of the five E15 replicate groups, in which EdU^+^ RGCs are more numerous. In the second LCM study, ∼26,000 RGCs were captured, and each replicate of the 10 E18 and three E15 groups contained up to 2000 EdU^+^/Brn3a^+^ RGCs. After LCM, microdissected cell samples were stored at −80°C until RNA extraction.

### RNA extraction and reverse transcription

RNA extraction was performed using the Qiagen RNeasy FFPE kit and RNA was converted to cDNA and pre-amplified for qPCR using the Qiagen RT^2^ PreAMP cDNA synthesis kit. In the initial qPCR experiment, because of the smaller than expected amount of RNA collected, RNA samples could not be quantified to ensure there was equal RNA input amounts in the reverse transcription reaction for each group, thus qPCR data were normalized only to the geometric mean of the reference genes *PPIA* and *RPL10A* (Becker et al., 2010). These genes showed a close pattern of expression changes across the groups. In the follow-up LCM study, in which a greater number of RGCs was captured, the concentration and integrity of RNA could be quantified using the Agilent RNA 6000 Pico Kit run on an Agilent 2100 Bioanalyzer prior to housekeeper normalization in qPCR; 1 ng total RNA from each sample was reverse transcribed. Although there was still RNA degradation (RNA Integrity Numbers < 8.0) from fixation and the unavoidable and extensive tissue processing prior to laser capture, the amount of RNA was sufficient to perform cDNA synthesis and qPCR. In both the pilot and second follow-up experiment, cDNA was pre-amplified using the Qiagen RT^2^ PreAMP cDNA synthesis kit for eight cycles.

#### qPCR

qPCR was performed using Custom Qiagen RT^2^ Profiler PCR Arrays and Qiagen RT^2^ SYBR Green ROX FAST Mastermix. Custom PCR arrays contained primers for 90 genes of interest (Table 1) and three housekeeping genes and three controls to verify PCR performance. A Corbett Rotor-Gene 6000 cycler was programmed with the following cycling settings: 95°C for 10 min, followed by 60 cycles of 95°C for 15 s, and 60°C for 30 s. Fluorescence threshold was fixed within the exponential phase of amplification and was identical for each run. A melting program (0.5°C stepwise from 60 to 95°C) was run after cycling and samples with multiple or abnormal melt peaks or those that crossed the threshold after cycle 45 were excluded from analysis.

**Table 1:** Gene primers in the Qiagen Custom RT^2^ Profiler PCR Array CAPR11291R

Gene	Function	RefSeq no.	FC
*Adcyap1*	Stimulates cAMP production; upregulated over development (Boeshore et al., 2004; Wang et al., 2007)	NM_016989	-
*Akt1*	Downstream of PI3K; critical in neurotrophin-mediated growth and survival (Kaplan and Miller, 2000; Perkinton et al., 2002; Campos et al., 2003)	NM_033230	↑
*Akt3*	Downstream of PI3K; critical in neurotrophin-mediated growth and survival (as above)	NM_031575	↕
*ApoE*	Involved in CNTF signaling, regulated over development (Fischer, 2012)	NM_138828	↓
*Arg1*	Downstream from cAMP in survival and regeneration; critical in overcoming myelin inhibition; regulated over development (Cai et al., 2002; Moore and Goldberg, 2011)	NM_017134	-
*ATF3*	Transcription factor; interacts with CREB and c-JUN; upregulated after injury, promotes regeneration (Seijffers et al., 2006; Moore and Goldberg, 2011)	NM_012912	-
*Bad*	Pro-apoptotic protein (Isenmann et al., 2003; Wilson and Di Polo, 2012)	NM_022698	-
*Bax*	Pro-apoptotic protein (as above)	NM_017059	↑
*Bcl2*	Anti-apoptotic protein; necessary for neuronal survival; overexpression reduces PCD (Brunet et al., 2001; Campos et al., 2003)	NM_016993	<2
*Bcl2l1*	Anti- or pro-apoptotic protein depending on splice variant (as above)	NM_031535	-
*Bcl2l11*	Pro-apoptotic protein; downstream of FOXO transcription factors (as above)	NM_022612	-
*BDNF*	Promotes RGC survival; axon branching; synaptogenesis (Numakawa et al., 2010)	NM_012513	↑
*CAMK1*	Phosphorylates CREB; prolongs ERK activation; regulates axon growth (Lonze and Ginty, 2002; Perkinton et al., 2002; Jiao et al., 2005)	NM_134468	↓
*CAMK2d*	Phosphorylates CREB; prolongs ERK activation; regulates axon growth and dendritic architecture; involved in depolarization-induced survival (as above)	NM_012519	↕
*CAMK2g*	Phosphorylates CREB; prolongs ERK activation; regulates axon growth, dendritic architecture, neurite length; involved in depolarization-induced survival (as above)	NM_133605	↓
*Casp8*	Apoptotic factor; expressed after injury; mediates apoptosis through FOXO1 (Brunet et al., 2001; O’Driscoll et al., 2006; Johnson et al., 2009)	NM_022277	-
*Casp9*	Apoptotic factor; activates effector caspases; involved in PCD (as above)	NM_031632	-
*Cdc42*	Rho family GTPase; positive regulator of actin dynamics, microtubule formation, growth cone formation, and axon elongation; acts in opposition to RhoA (Zhou and Snider, 2006; Major and Brady-Kalnay, 2007; Hausott et al., 2009)	NM_171994	↑
*Cdh1*	Adhesion factor; regulates axon growth and patterning (Konishi et al., 2004)	NM_031334	-
*CNTFR*	Receptor for CNTF; promotes growth and survival (Yip and So, 2000; Lingor et al., 2008)	NM_001003 929	↑
*CREB1*	Transcription factor; critical for survival and growth signaling (Lonze and Ginty, 2002; Moore and Goldberg, 2011)	NM_031017	↑
*DCC*	Guidance factor; receptor for netrin-1; involved in guidance toward optic disk (Koeberle and Bähr, 2004; Erskine and Herrera, 2007)	NM_012841	-
*DCX*	Microtubule-associated protein; growth promoting (Blackmore et al., 2010)	NM_053379	-
*DSCAM*	Guidance factor; involved in self-avoidance (Fuerst et al., 2008,2009)	NM_133587	<2
*EFNA2*	Ephrin A2 ligand; involved in inhibition; expressed in development and after axotomy in adult (O’Leary and Wilkinson, 1999; Cang et al., 2008)	NM_001168 670	-
*EGFR*	Receptor for EGF; involved in myelin inhibition; required for development of some CNS regions (Hannila and Filbin, 2008; Hausott et al., 2009)	NM_031507	-
*ELK1*	Transcription factor; regulates cell cycling; pro-survival effects (Hausott et al., 2009)	XM_001055 949	-
*EphA4*	Receptor for Ephrin A and B; involved in inhibition (O’Leary and Wilkinson, 1999; Reber et al., 2004; Lindqvist et al., 2010)	NM_001162 411	<2
*Fos*	Involved in regulation of TrkB expression (Chang et al., 2004; Park et al., 2004)	NM_022197	<2
*FOXO3*	Pro-apoptotic transcription factor; inhibited by neurotrophin signaling via Akt and SGK (Brunet et al., 2001)	NM_001106 395	-
*Gap43*	Protein required for axon growth (Schaden et al., 1994)	NM_017195	-
*GHR*	Growth hormone receptor; pro-survival effects on developing RGCs via CREB (Sanders et al., 2008)	NM_017094	↓
*GSK3B*	Influences cytoskeleton assembly; involved in pro-apoptotic signaling; apoptotic function inhibited by Akt (Brunet et al., 2001; Tokuoka et al., 2002; Alabed et al., 2010)	NM_032080	↑
*HAND1*	Transcription factor, required for sympathetic neuronal survival (Doxakis et al., 2008)	NM_021592	<2
*HAND2*	Transcription factor, required for sympathetic neuronal survival (as above)	NM_022696	-
*IGF-1R*	Growth factor receptor; promotes RGC survival and growth (Goldberg et al., 2002)	NM_052807	↕
*Il6R*	Cytokine receptor; promotes RGC survival and growth (Cao et al., 2006; Moore and Goldberg, 2011)	NM_017020	↑
*IL6st*	Part of the IL6R complex; promotes RGC survival and growth (as above)	NM_001008 725	↓
*JAK1*	Component of JAK/STAT signaling cascade; involved in signaling via IL6R, CNTFR, and GHR; regulated in development (Park et al., 2004; Wang et al., 2007)	NM_053466	<2
*JAK2*	Component of JAK/STAT signaling cascade (Park et al., 2004)	NM_031514	-
*Jun*	Transcription factor; involved in apoptosis, growth, and regeneration (Lu et al., 2003; Tedeschi, 2012)	NM_021835	-
*KLF4*	Transcription factor; inhibits axon growth, regulated in development (Moore et al., 2009)	NM_053713	<2
*KLF6*	Transcription factor; promotes axon growth (Veldman et al., 2007; Moore et al., 2009)	NM_031642	-
*KLF7*	Transcription factor; promotes axon outgrowth (as above)	NM_001108 800	↕
*KLF9*	Transcription factor; inhibits axon outgrowth (as above)	NM_057211	-
*L1CAM*	Cell adhesion molecule, upregulated in axon regeneration (Fischer et al., 2004)	NM_017345	-
*LIFR*	IL6 class cytokine receptor; neurotrophic factor; promotes survival and growth in RGCs (Goldberg et al., 2002; Moore and Goldberg, 2011; Fischer, 2012)	NM_031048	-
*LINGO1*	Part of Nogo receptor complex (Mi et al., 2004; Fu et al., 2008; Hannila and Filbin, 2008)	NM_001100 722	-
*MafK*	Transcription factor; upregulated after axotomy (Fischer et al., 2004)	NM_145673	-
*MAP2k1*	Major neurotrophin signaling pathway (Atwal et al., 2000; Gao et al., 2003; Hausott et al., 2009; Johnson et al., 2009)	NM_031643	-
*MAP2k2*	Major neurotrophin signaling pathway (as above)	NM_133283	↕
*MAPK1*	Kinase involved in MAPK signaling (Kaplan and Miller, 2000; Reichardt, 2006)	NM_053842	<2
*MAPK14*	Kinase involved in both apoptosis and survival signaling; downstream phosphorylation of CREB; possibly involved in neurite outgrowth (as above)	NM_031020	-
*MAPK3*	Kinase involved in MAPK signaling (as above)	NM_017347	↑
*MAPK8*	Kinase involved in pro-apoptotic signaling; inhibits Akt, downstream in p75NTR apoptotic signaling; activates c-Jun; regulates microtubule formation (Kaplan and Miller, 2000; Roux and Barker, 2002; Zhou and Snider, 2006)	XM_341399	<2
*MAPK9*	Kinase involved in pro-apoptotic signaling; involved in developmental apoptosis (Kuan et al., 1999; Le-Niculescu et al., 1999)	NM_017322	<2
*mTOR*	Kinase; controls protein synthesis required for axon growth; downstream of PI3K (Park et al., 2008)	NM_019906	↓
*NFATc3*	Transcription factor; involved in axonal growth; neurotrophins alter activity (Zhou and Snider, 2006; Moore and Goldberg, 2011)	NM_001108 447	<2
*NFATc4*	Transcription factor; involved in axonal growth; neurotrophins alter activity (as above)	NM_001107 264	<2
*Ngfr*	p75NTR; low-affinity neurotrophin receptor; pro-neurotrophin receptor; pro- or anti-apoptotic depending on conditions (Kaplan and Miller, 2000; Roux and Barker, 2002)	NM_012610	-
*Nptx2*	Role in synaptic plasticity (Fischer et al., 2004)	NM_001034 199	-
*Nrxn1*	Involved in synapse formation (Barker et al., 2008)	NM_021767	↕
*Nrxn2*	Involved in synapse formation (as above)	NM_053846	-
*Ntrk2*	TrkB; receptor for BDNF (Atwal et al., 2000; Kaplan and Miller, 2000)	NM_012731	<2
*Optn*	Involved in axonal transport, regulated in development (Wang et al., 2007)	NM_145081	↑
*Pde4b*	Enzyme responsible for cAMP hydrolysis; inhibited by neurotrophin signaling (Gao et al., 2003; Hannila and Filbin, 2008)	NM_017031	-
*PKCcd*	Kinase involved in PI3K and p75NTR/NgR signaling; regulates MAPK activity (Reichardt, 2006; Zhou and Snider, 2006; Major and Brady-Kalnay, 2007)	NM_133307	-
*PLCg1*	Phospholipase activated through TrkB; extends MAPK signaling; increases intracellular Ca^2+^ (Segal, 2003; Zhou and Snider, 2006; Numakawa et al., 2010)	NM_013187	↓
*POU4F1*	Transcription factor Brn3a; involved in dendritic branching and architecture (Weishaupt et al., 2005; Badea et al., 2009; Voyatzis et al., 2012)	XM_341372	↓
*POU4F2*	Transcription factor Brn3b; involved in cell-fate determination; critical for RGC differentiation, involved in axonal development (as above)	NM_134355	-
*Psip1*	Transcription factor; regulates expression of growth-associated genes; involved in dendritic arborization (Zhao et al., 2008)	NM_175765	-
*PTEN*	Phosphatase; converts PIP_3_ to PIP_2_; inhibits Akt and downstream signaling (Park et al., 2008)	NM_031606	↑
*Ptk7*	Transcription factor; downstream in PI3K and MAPK pathways; involved in BDNF-induced survival (Chang et al., 2004)	NM_001106 889	-
*Rac1*	Rho family GTPase; involved in growth cone mechanics; inhibits RhoA; involved in nasal-temporal crossing (Pearse, 2004; Schiller, 2006; Major and Brady-Kalnay, 2007)	NM_134366	↑
*RelA*	NFkB transcription factor; involved in axonal growth and survival; activated by p75NTR (Moore and Goldberg, 2011)	NM_199267	-
*RhoA*	GTPase; activates ROCK triggering growth cone collapse; binds to p75NTR (Park et al., 2005; Lingor et al., 2008; Mi, 2008; Hausott et al., 2009)	NM_057132	↑
*Rock1*	Kinase; downstream of RhoA in mediating growth cone collapse (as above)	NM_031098	-
*Rps6*	Ribosomal protein; growth and survival promoting (Sun and He, 2010)	NM_017160	-
*Rps6ka1*	Phosphorylates ribosomal Protein s6; activates STATs, CREB; involved in MAPK survival signaling (Lonze and Ginty, 2002; Chang et al., 2004; Koeberle and Bähr, 2004)	NM_031107	-
*Rps6ka2*	Phosphorylates ribosomal Protein s6; activates STATs, CREB; involved in MAPK survival signaling (as above)	NM_057128	<2
*Rps6kb1*	Phosphorylates ribosomal Protein s6; downstream of Akt and mTOR (Manning and Cantley, 2007; Park et al., 2008)	NM_031985	-
*Rtn4r*	Part of NgR-p75NTR signaling complex; mediates inhibitory signaling dependent on cAMP levels and Arg1 activity (Cai et al., 2002; Schwab, 2004; Fu et al., 2008)	NM_053613	-
*SGK1*	Kinase; involved in PI3K pathway, similar to Akt; inhibits pro-apoptotic factors, e.g., FOXO3 (Brunet et al., 2001)	NM_019232	-
*SOCS3*	Negative regulator of JAK/STAT signaling; knock-out improves regeneration (Sun and He, 2010; Hellström et al., 2011; Fischer, 2012)	NM_053565	-
*Sort1*	Pro-neurotrophin receptor; complexes with p75NTR; pro-apoptotic (Jansen et al., 2007; Teng et al., 2010)	NM_031767	<2
*SOX11*	Transcription factor; increases expression of growth-associated molecules (Veldman et al., 2007; Moore and Goldberg, 2011)	NM_053349	-
*STAT1*	Activated by STAT3 via JAK/STAT cascade (Moore and Goldberg, 2011; Tedeschi, 2012)	NM_032612	↑
*STAT3*	Component of JAK/STAT signaling cascade; pro-survival signaling (as above)	NM_012747	↑
*Stk24*	Kinase; critical for axon regeneration in cultured CNS neurons (mst3B; Lorber et al., 2009)	NM_001127 494	↑
*Tnfrsf19*	TROY; takes place of p75NTR with LINGO and NgR (Park et al., 2005; Hisaoka, 2006; Mi, 2008)	Unigene: Rn.202731	-
*PPIA*	Housekeeping gene (commonly used rat housekeeping gene in laboratory)	NM_017101	-
*Rpl10a*	Housekeeping gene (Hong Cai et al., 2007)	NM_031065	-
*HPRT*	Housekeeping gene (Vázquez Chona and Vazquez, 2004)	NM_012583	-

Brief function, relevant citations, and RefSeq number are listed for each gene. Arrows in the right column indicate a >±2 relative fold change (FC) in gene expression when comparing expression levels between E18/P1 and E15/P0, E18/P1 and E18/P5, or E18/P5 and E15/P0 groups. ↕, relative change in expression, either up or down, depending on pairwise comparison; see Fig. 2B for details. <2, genes with a change in expression <±2; -, genes that failed to amplify in one or more samples, or amplification was evident but only after cycle 45 and therefore discounted.

### Statistical analysis

In both the first and second qPCR rounds, expression of each gene between conditions was compared using the ΔCt method (Livak and Schmittgen, 2001; Pfaffl, 2001; Schmittgen and Livak, 2008). In both rounds, two reference genes (*PPIA* and *RPL10a*) were used to normalize expression across groups; the third (HPRT) was not used because of excessive variance. For each gene of interest, comparisons of gene expression across the conditions were performed: growing versus target-dependent RGCs, growing versus transitional RGCs, and transitional versus target-dependent RGCs. For each pairwise comparison, a fold change value was calculated to indicate relative gene expression level and a Student’s *t* test was performed. A *p* value <0.05 was considered statistically significant, and only fold changes >±2 were considered. Data are presented as mean fold change.

### Discriminant analysis

To further assess changes in gene expression between groups in the second LCM study, we used discriminant analysis, a multivariate statistical technique used for differentiating groups using multiple quantitative variables. Using JMP software, we calculated two canonical scores that represented all of the variability of the dataset. The canonical scores were then compared between groups to identify significant differences using ANOVA. Differences in individual gene expression were then determined using ANOVA and Tukey *post hoc* tests.

A first-pass analysis included expression profiles of all 45 genes that were successfully amplified to determine whether there were significant differences between the groups of RGCs at different developmental stages. Having established differences between the groups in this global analysis, we then performed a more focused analysis to look for differential activation of individual pathways at the different developmental stages. We examined genes implicated in selected pathways related to BDNF signaling as follows: downstream of TrkB via PLCg (BDNF, CAMK1, CAMk2d, CAMk2g, CREB, and PLCg1) and downstream of TrkB via Ras (BDNF, CREB, MAP2k2, MAPk1, and MAPk3). We also examined pathways implicated in neuronal survival and axon elongation: Akt signaling (Akt1, Akt3, Bax, BDNF, and GSK3B) and JAK-STAT signaling (CNTFRa, JAK2, STAT1, and STAT3).

### *In vitro* analysis of trophic dependence

Nine time-mated female Wistar rats were injected intraperitoneally at E15 (*n* = 2) or E18 (*n* = 7) with BrdU, 50mg/kg maternal body weight, three times during the day (at 9 A.M. and 1and 5 P.M.). At P1, pups were killed with sodium pentobarbital (Lethabarb) and eyes were removed. Retinas were dissected from eyes and pooled into dPBS, then dissociated using the MACS neural dissociation kit (Miltenyi Biotec) according to manufacturer’s instructions. RGCs were isolated using the MACS RGC Isolation kit (Miltenyi Biotec), following standard depletion and selection protocols. Cells were resuspended in defined serum-free growth medium (Ullian et al., 2004; modified from Bottenstein and Sato, 1979). Neurobasal media contained B27 supplement, triiodothyronine, transferrin, progesterone, sodium selenite, n-acetyl cysteine (Sigma-Aldrich), 2 mm l-glutamine, 1 mm sodium pyruvate (Life Technologies), 5 μg/ml insulin, and 10 μm forskolin (Sigma), with or without 50 ng/ml BDNF (PeproTech). RGCs were seeded onto poly-d-lysine (70 kDa, 10 μg/ml; Sigma) and mouse type-1 laminin (Sigma)-coated 8-well culture slides (Falcon; BD).

Purified RGC populations were fixed after 1 d or 2 d in culture (analogous to P2 and P3 *in vivo*). For immunostaining, cells were fixed with 4% paraformaldehyde, washed, and double labeled with anti-β-III tubulin antibody (TUJ1 clone, rabbit; Covance PRB-435P, 1:4000) with goat anti rabbit Cy3 secondary (Jackson ImmunoResearch 111166047, 1:400), both for 30 min at room temperature, followed by 30 min 2 m HCl treatment for 30 min at 37°C. Cells were then incubated overnight with BrdU antibody (mouse; Roche 11170376001, 1:100) in diluent containing 4% NGS, 3% BSA, and 0.3% Triton X-100, washed, and incubated for 2 h at room temperature with goat anti-mouse FITC secondary antibody (Cappel 55521; 1:100). Slides were coverslipped in Dako Fluorescent mounting medium.

Data were obtained from four separate culture preparations of purified E15 RGCs and five preparations of purified E18 RGCs. Two wells were counted for each culture for each condition (±BDNF, 24 h or 48 h survival). Slides were examined by light and multichannel fluorescent microscopy using an Olympus BX50 microscope equipped with a motorized stage (*x*,*y*,*z*), and a digital, color top-mounted camera (QImaging), and linked to a computer with Stereo-Investigator software (V10.0; MBF Bioscience). Each well of the culture slide was outlined at its border using a 4× objective (bright field) using the contour mapping feature in the Stereo-Investigator software. The sampling area consisted of 100 uniform but random counting frames (550 × 330 μm), which were calculated using the Systematic Random Sampling layout feature (total area sampled ∼18 mm^2^, ∼25% of the surface area). All β-III-tubulin^+^ cells within each frame were counted using fluorescent light (TRITC filter) and classified according to either the presence or absence of prominent β-III-tubulin^+^ processes. Incorporation of BrdU into RGC nuclei was classified subjectively by a single operator and an assessment made of the nuclear area containing BrdU (either no BrdU, <50% of nuclei area expressing BrdU, or >50%). Each β-III-tubulin^+^ RGC was therefore counted and classified based on the presence of absence of processes and BrdU content within the nucleus. Results were tallied by the software and exported to Microsoft Excel for further analysis. Data were quantified from four E15 and 5 E18 cultures (two wells counted for each culture for each condition).

To examine BDNF expression in late-born RGCs, in two additional purified RGC cultures from litters labeled at E18 with BrdU, RGCs were maintained for 24 h or 48 h in Neurobasal media without recombinant BDNF. At 24 h or 48 h, cultures were fixed in 4% paraformaldehyde, and after blocking (10% NGS, 0.2% Triton X-100 for 30 min) cultures were immunostained with antibodies to BDNF (rabbit; BiosensisR-172-20 or Santa Cruz Technology SC-546) or β-III-tubulin (Covance PRB-435P, 1:400) in blocking buffer for 1 h followed by incubation in secondary antibodies (1 h, anti-rabbit Cy3). Cultures were then reacted for BrdU as described above and coverslipped.

### Anti-BDNF antibody specificity

To confirm that both the BDNF antibodies bound specifically to BDNF we performed an absorption control test using recombinant BDNF protein (Human BDNF; PeproTech). First, BDNF protein was eluted in 2× Laemmli buffer [250 mm Tris, 10% (v/v) glycerol, 4% (w/v) SDS, 2% (v/v) β-mercaptoethanol, 0.005% (w/v) bromophenol blue (Sigma-Aldrich)] in double-deionized water (DDW; pH 6.8), separated by SDS-PAGE (Mini-PROTEAN TGX Stain-Free Precast Gels; Bio-Rad) and transferred to nitrocellulose membrane (Trans-Blot Turbo Mini Nitrocellulose Transfer Pack; Bio-Rad). Recombinant proteins were detected by Western blot using 3% (w/v) skim milk powder in TBS-T [100 mm Tris, 154 mm NaCl, 0.1% (v/v) Tween 20, in DDW, pH 7.5] blocking buffer, antibodies to BDNF (rabbit; Biosensis R-172-20, 1:500 or Santa Cruz Biotechnology, SC-546; 1:500) and α-rabbit-HRP secondary antibody (1:10,000 dilution; Pierce Scientific). Proteins were visualized by chemiluminescence (Immun-Star; Bio-Rad) using the ChemiDoc system (Bio-Rad). In the absorption control, the anti-BDNF antibody (1 µg, rabbit; Biosensis R-172-20) was incubated with 10 μg of BDNF diluted in 100 µl blocking agent and incubated overnight at 4°C. The pre-absorbed primary antibody was then used to immunostain purified RGC cultures using the protocol described above.

## Results

### qPCR of laser-captured RGCs before, during, and after target innervation

**Figure 1:**
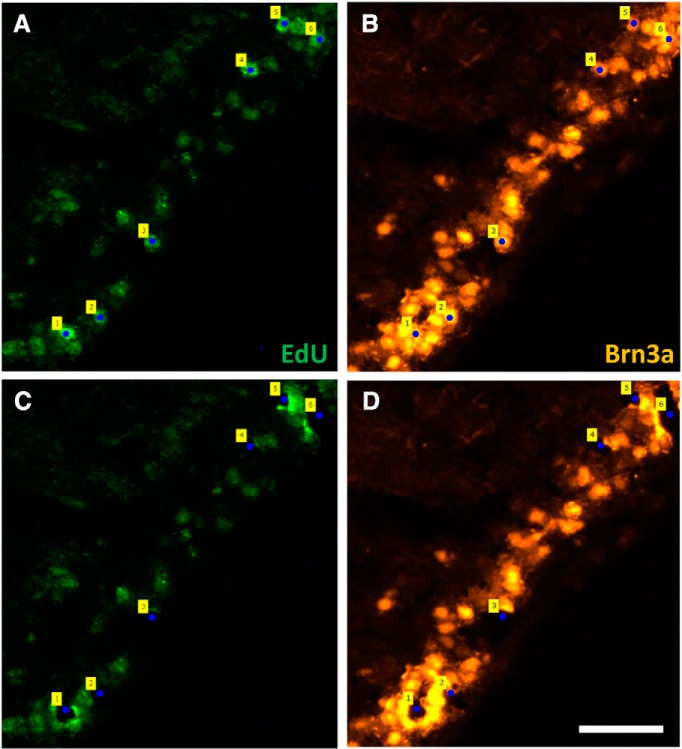
LCM isolates individual RGCs from tissue sections; neurons are identified by their birthdate. EdU and Brn3a-stained retinal section from a pup that received an E18 EdU injection and was perfused at P1. ***A***, ***B***, Section before LCM stained for EdU (***A***) and Brn3a (***B***); DL RGCs were tagged (numbers). ***C***, ***D***, Section after LCM, stained for EdU (***C***) and Brn3a (***D***); tags show spaces left following catapulting of DL cells. Scale = 50μm.

In retinas used for LCM, EdU^+^ cells were seen in the GCL and other layers of the retina. In E15-injected EdU groups, EdU^+^ cells in the GCL were found in all regions of the retina, whereas in E18 EdU groups there were far fewer double-labeled (DL) EdU^+^/Brn3a^+^ neurons. These RGCs were mostly located in the periphery, consistent with previous reports (Reese and Colello, 1992; Dallimore et al., 2002). LCM using the PALM DuoFlex CombiSystem successfully catapulted individual cells from retinal sections. Figure 1 shows retinal sections stained for EdU and Brn3a, with DL cells tagged before and after laser catapulting.

 Two separate LCM studies were performed. In the initial pilot experiment we captured almost 9000 RGCs, giving at least 800 RGCs per replicate group; however, with this number of RGCs RNA and cDNA yields were below the sensitivity of the NanoDrop and Agilent Bioanalyzer 6000 Nano Kits. Nonetheless we proceeded to qPCR analysis of the entire cDNA stock obtained in this first experiment. Several genes exhibited at least a fourfold change in mRNA expression levels between target-dependent and growing RGCs, although due to failure of amplification in some samples none of these changes reached statistical significance. Genes encoding BDNF, NFATc3, Adcyap1, EphA4, and STAT3 all appeared to be upregulated in growing RGCs compared with target-dependent RGCs, whereas ApoE and NFATc4 were downregulated. Importantly, BDNF transcript levels exhibited by far the greatest reduction between growing and target-dependent RGCs, consistent with PCR data from the second LCM study.

**Figure 2:**
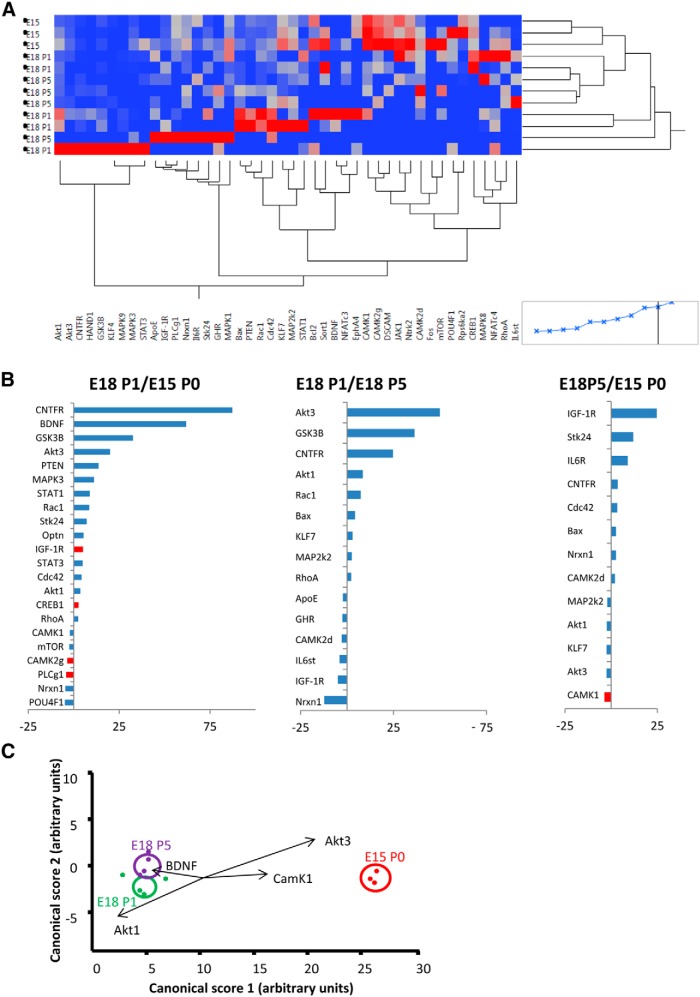
***A***, Heat map and cluster analysis for all genes with reliable PCR amplification. ***B***, Pairwise comparisons between actively growing (E15/P0), target-independent (E18/P1), and transitional (E18/P5) RGCs, showing all genes with >±2-fold change. Significant differences are shown in red. ***C***, Plots show canonical scores 1 (*x*-axis) and 2 (*y*-axis) from a multivariate discriminant analysis of overall gene expression levels (2^-ΔCT^). The two canonical scores represent 100% of the variance. Axes represent arbitrary units of SD. Circles represent the 95% confidence region to contain the true mean of the treatment groups. Black lines show the coordinate direction (for simplicity, only selected individual gene expression levels are shown here) in canonical space. Note that the length of the lines is not representative of effect size due to the multidimensional nature of the analysis. Comparisons are made on canonical scores using ANOVA with Tukey’s *post hoc* test: E15/P0 versus E18/P1: *p* < 0.0001 (Canonical 1); E15/P0 versus E18/P5: *p* < 0.0001 (Canonical 1); E18/P5 versus E18/P1: *p* = 0.0203 (Canonical 2).

The initial LCM work was needed to establish optimal protocols for identifying gene changes in individually isolated postnatal RGCs. Based on this first study, we next used LCM to accumulate approximately three times as many RGCs (25,871 in total). In this second experiment only Brn3a^+^ EdU^+^ RGCs were selected for capture. Up to 2000 RGCs were used in each replicate qPCR group. In the second laser-capture experiment, of the initial 90 genes selected for amplification (Table 1), 45 genes were consistently amplified in sufficient groups to allow for statistical comparison. These were as follows: Akt1, Akt3, ApoE, Bax, Bcl2, BDNF, CAMK1, CAMK2d, CAMK2g, Cdc42, CNTFRa, CREB1, DSCAM, EphA4, Fos, GHR, GSK3B, HAND1, IGF-1R, Il6R, IL6st, JAK1, KLF4, KLF7, MAP2k2, MAPK1, MAPK3, MAPK8, MAPK9, mTOR, NFATc3, NFATc4, Nrxn1, Ntrk2, Optn, PLCg1, POU4F1, PTEN, Rac1, RhoA, Rps6ka2, Sort1, STAT1, STAT3, Stk24 (Fig. 2*A*).

Growing RGCs showed altered expression of a significant number of genes when compared with either target-dependent or transitional RGCs. The 30 genes with a >±2-fold change in expression between the various groups are displayed in Figure 2*B*. Comparison between growing and target-dependent RGC groups revealed a statistically significant relative increase in expression of IGF-1R and CREB1, and a significant relative decrease in expression of CAMK2g and PLCg mRNAs in growing RGCs. Comparatively large fold increases in mRNA levels in growing RGCs were also noted for CNTFRa, BDNF, GSK3B, Akt3, and PTEN. Fewer genes differed when comparing growing versus transitional, and transitional versus target-dependent, RGC groups. CAMK1 expression was significantly lower in transitional compared with target-dependent RGCs (Fig. 2*B*), but overall the extent of any fold change was generally less in these comparisons. Exceptions were large fold increases in Akt3, GSK3B, and CNTFRa gene expression and a more than 10-fold decrease in neurexin 1 gene expression in growing versus transitional RGCs, and increased expression of IGF-1R, Stk24, and IL6R in transitional versus target-dependent RGCs.

### Discriminant analysis and significant differences between growing and target-dependent RGCs

To further assess changes in gene expression between groups, we used discriminant analysis, a multivariate statistical technique used for differentiating groups using multiple quantitative variables. Discriminant scores confirmed that all replicates could be allocated with 100% confidence to their appropriate group/developmental stage based on the pattern of gene expression. An ANOVA comparing the canonical scores revealed significant differences between conditions and Tukey *post hoc* tests confirmed that each condition was significantly different from the other two (growing vs target-dependent *p* < 0.0001; transitional vs target-dependent: *p* < 0.0001; growing vs transitional: *p* = 0.023; Fig. 2*C*). Genes that contributed most to these differences were BDNF, CAMK1, Akt1, and Akt3.

### Pathway analysis and intracellular signaling

**Figure 3:**
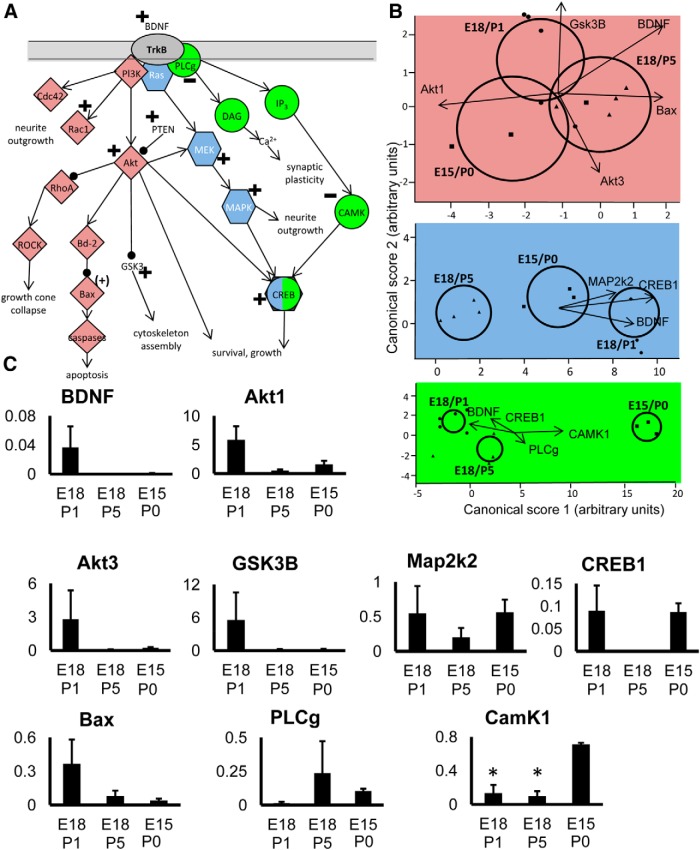
Analysis of genes in pathways downstream of BDNF signaling. ***A***, Diagram showing the main pathways analyzed. Note that some genes in the diagram did not show changes, or were not analyzed, but are included for context. ***B***, Plots show canonical scores 1 (*x*-axis) and 2 (*y*-axis) from a multivariate discriminant analysis of a subset of genes within specific pathways downstream of BDNF. Colors are used to identify distinct pathways, but in some cases genes are common to more than one pathway. The two canonical scores represent 100% of the variance. Axes represent arbitrary units of SD. Circles represent the 95% confidence region to contain the true mean of the treatment groups. Black lines show the coordinate direction (for simplicity, only selected individual gene expression levels are shown here) in canonical space. Note that the length of the lines is not representative of effect size due to the multidimensional nature of the analysis. Comparisons are made on canonical scores using ANOVA with Tukey’s *post hoc* test: via PlCg: E15/P0 versus E18/P1: *p* < 0.0001; E15/P0 versus E18/P5: *p* < 0.0001; E18/P5 versus E18/P1: *p* < 0.0016. Via Ras: E18/P1 versus E18/P5: *p* < 0.0001; E15P0 versus E18/P5: *p* = 0.0008; E18/P1 versus E15/P0: *p* = 0.0026. Via GSK3b: E18/P5 versus E15/P0: *p* = 0.0483; E18/P5 versus E18/P1: *p* = 0.1186; E18/P1 versus E15/P0: *p* = 0.6579. ***C***, Histograms showing mean relative expression level of individual genes (±SEM) for each group. Significant differences are indicated with an asterisk.

**Figure 4:**
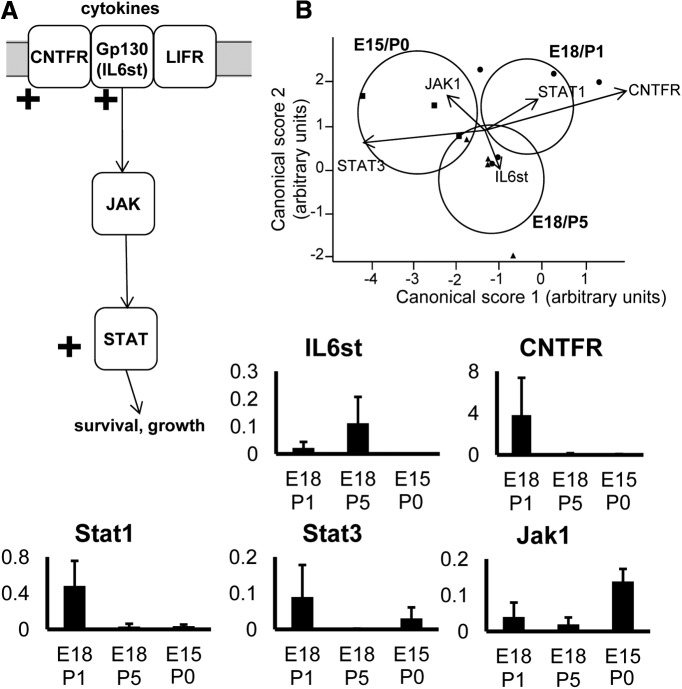
Analysis of genes in pathways downstream of CNTF signaling. ***A***, Diagram showing the main pathways analyzed. Note that some genes in the diagram did not show changes, or were not analyzed, but are included for context. ***B***, Plots show canonical scores 1 (*x*-axis) and 2 (*y*-axis) from a multivariate discriminant analysis of a subset of genes downstream of CNTF. The two canonical scores represent 100% of the variance. Axes represent arbitrary units of SD. Circles represent the 95% confidence region to contain the true mean of the treatment groups. Black lines show the coordinate direction (for simplicity, only selected individual gene expression levels are shown here) in canonical space. Note that the length of the lines is not representative of effect size due to the multidimensional nature of the analysis. Comparisons are made on canonical scores using ANOVA with Tukey’s *post hoc* test: E18/P1 versus E15/P0: *p* = 0.0177; E18/P5 versus E15/P0: *p* = 0.1261; E18/P1 versus E18/P5: *p* = 0.4446.

To further examine the potential involvement of signaling pathways in RGCs during the transition to target dependence, discriminant analysis was performed on genes known to be associated with particular growth or survival-associated pathways, focusing on systems downstream of BDNF and TrkB (Fig. 3), and the CNTF receptor complex (Fig. 4).

BDNF signaling via TrkB activates three distinct pathways, all partially covered by our gene arrays (Fig. 3*B*,*C*). Genes that are components of the CAMK downstream pathway (Fig. 3*A*,*B*, green) were significantly different between growing, transitional and target-dependent RGC groups. Genes in the MAPK downstream pathway (Fig. 3*A*,*B*, blue) were also significantly different, with expression of MAPK isoforms increased in growing compared with nongrowing RGCs. A significant difference in the expression profile of the Akt signaling pathway (Fig. 3*A*,*B*, pink) was seen, primarily attributed to an increase in expression of Akt isoforms in growing RGCs (Fig. 3*C*). Additionally, the growing and target-dependent RGC groups showed a significantly different gene expression profile for at least some factors involved in CNTF signaling, with a peak of CNTFRa and downstream Stat1 and Stat3 expression in actively growing, late-born RGCs (Fig. 4). Transcripts for IL6st (also known as gp130) also appear to be more prevalent, particularly in transitional RGCs.

### Tissue culture: RGC survival in the presence or absence of BDNF

**Figure 5:**
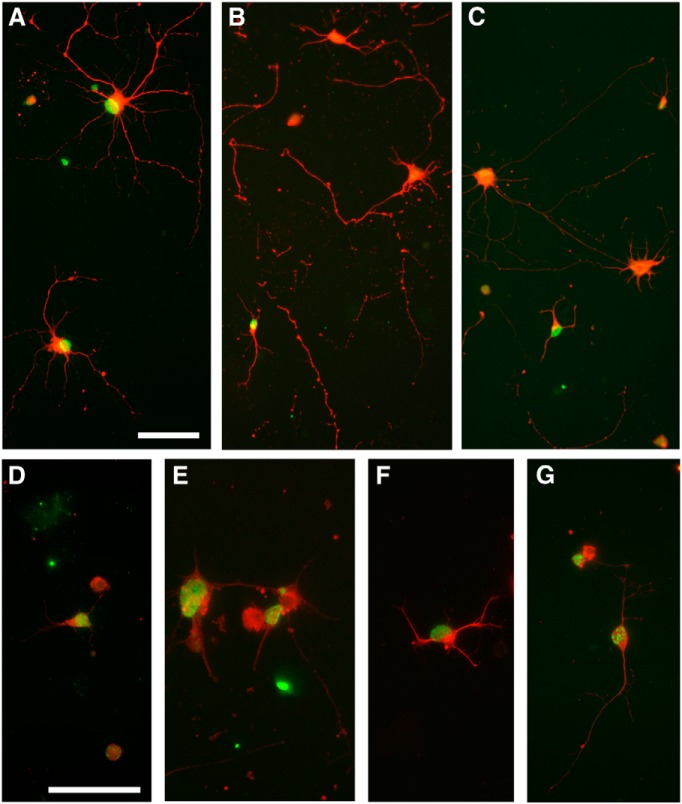
Examples of BrdU-labeled (green), β-III-tubulin^+^ (red) RGCs purified at P1 and examined after 48 h in culture in the presence (***A***) or absence (***B***, ***C***) of BDNF. ***A***, Process-bearing, BrdU^+^ E15 RGCs are evident at 48 h in the presence of BDNF. ***B***, ***C***, Absence of BDNF resulted in the overall loss of cultured RGCs and reduction in neurite expression; however, E18 RGCs continued to survive and many expressed processes. Note the fragmented neurites from loss of cells between 24 and 48 h. ***D–G***, E18 RGCs cultured without BDNF immunostained with a BDNF antibody. ***D***, Twenty-four hour culture. ***E–G***, Forty-eight hour culture. Scale bars: 50µm.

Data were obtained from four separate culture preparations of purified P1 RGCs previously labeled with BrdU on either E15 or E18. Each of the 72 wells was initially plated with ∼15,000 cells. Counting areas were randomly sampled (see Materials and Methods). Independent of the BrdU label, in total ∼35,000 β-III-tubulin^+^ RGCs were counted. Based on these counts we estimated that, in the presence of BDNF, on average 18.4 and 14.3% of initially plated RGCs remained viable at 24 and 48 h, respectively, compared with 11.4 and 6.2% in the absence of BDNF. Expression of clearly defined, multiple β-III-tubulin^+^ processes was also affected by neurotrophic support (Fig. 5); irrespective of birthdate, with BDNF 65.5 and 52.9% of surviving RGCs expressed processes at 24 and 48 h, compared with 41.5 and 33.4% in the absence of this neurotrophin.

**Figure 6:**
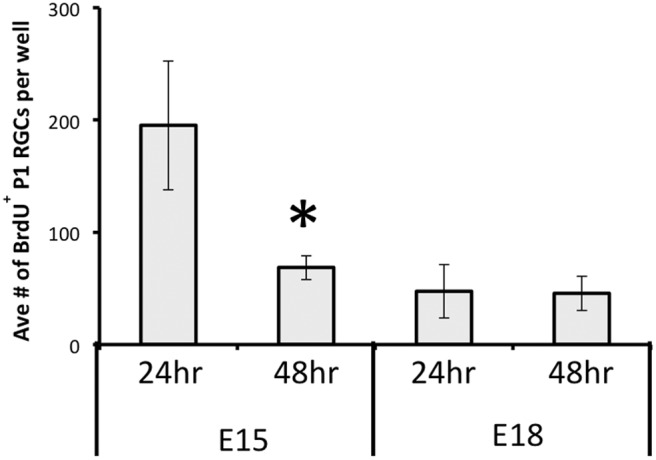
The mean number (±SEM) per well of E15 or E18 BrdU-labeled, β-III-tubulin immunopositive P1 RGCs after either 24 h or 48 h *in vitro*, in the absence of BDNF in the culture medium. Note the significant (asterisk, Mann–Whitney *U* test, *p* < 0.05) loss of E15 RGCs between 24 and 48 h, but the numbers of E18 RGCs remained unchanged.

On the critical issue of viability of RGCs identified by birthdate, we calculated the average number (±SEM) of E15 or E18 BrdU^+^ RGCs (BrdU label in >50% of the nucleus) in the presence or absence of BDNF at 24 and 48 h. There was no change in E18 RGC counts in the two conditions, indicating that these late-born cells did not require exogenous BDNF for survival; however, there was a significant (*Mann–Whitney *U* test, *p* < 0.05) loss of E15 RGCs between 24 and 48 hours (Fig. 6). Consistent with this, in addition to the overall loss of viable β-III-tubulin^+^ RGCs at 48 h, the proportion of these surviving β-III-tubulin^+^ RGCs that were born on E15 was decreased, and the proportion of E18 BrdU^+^ RGCs increased, in 48 h cultures that lacked BDNF (Table 2). Many surviving E18 RGCs continued to express processes (Fig. 5*B*,*C*), thus the distinction between early-born and late-born RGCs was even greater when only analyzing process-bearing RGCs. Without BDNF, between 24 and 48 h the proportion of surviving β-III-tubulin^+^ E15 RGCs in the cultures was reduced by almost half, whereas the proportion of E18 RGCs increased from 1.5 to 3.6% (Table 2).

**Table 2:** Proportion of surviving, birth-dated RGCs in the presence or absence of BDNF, 24 and 48 h after plating

	E15 BrdU		E18 BrdU	
	24 h	48 h	24 h	48 h
All β-III-tubulin ^+^ RGCs				
With BDNF	28.0%	28.2%	1.5%	2.3%
No BDNF	24.2%	16.9%	1.5%	3.3%
				
Process-bearing RGCs				
With BDNF	37.0%	38.8%	1.4%	2.8%
No BDNF	34.0%	20.0%	1.5%	3.6%

In the second series of *in vitro* studies, E18 BrdU-labeled RGCs were isolated and cultured for either 24 h or 48 h in the absence of BDNF. After fixation, RGCs were immunostained with antibodies to BDNF (Fig. 5*D–G*). Immunostaining was substantially reduced when antibodies were pre-absorbed with recombinant BDNF protein (data not shown). Although BDNF positivity was not entirely restricted to the BrdU^+^ population (many surviving neurons would be from outside the labeling window), BrdU^+^ RGCs were immunopositive for BDNF, with qualitatively greater staining at 48 h compared with 24 h.

## Discussion

In the developing rat, a significant proportion of RGCs die at the time their axons are growing into and innervating central target sites such as the SC (Dallimore et al., 2002, 2010). Using custom qPCR arrays to quantify gene expression in birth-dated neonatal RGCs isolated by LCM, we have, for the first, time obtained snapshots of gene expression in maturing CNS neurons *in situ*. This novel methodology allowed an unprecedented investigation of the expression status of identified RGCs, at different stages of maturation *in vivo*, without confounding issues associated with analysis of whole retinal tissue or analysis of gene expression *in vitro* on cultured or cell-sorted RGCs. We have identified subtle differences in gene expression in RGCs that (1) are growing independently toward their target, (2) have axons in the process of target ingrowth, and (3) have completed ingrowth and now require central targets for trophic support. These differences, discussed in detail below, include the downregulation of endogenous BDNF expression as RGCs innervate central targets, along with changes in expression of genes involved in signaling pathways downstream of TrkB. There were also maturational changes in responsiveness to cytokines and other growth factors. Although further work is needed to validate and clarify these results, the approach utilized in this study should eventually assist in unraveling the complex factors involved in circuit formation during visual system development.

### The switch to target dependency in developing RGCs: BDNF

We obtained two lines of evidence suggesting that RGCs switch to a particular dependency on target-derived BDNF as their axons enter retinorecipient sites in the brain. The first comes from our qPCR array laser-capture experiments, both of which demonstrated that BDNF mRNA expression in growing late-born RGCs was considerably greater than in RGCs with axons already in central targets. While previous work has shown that BDNF is expressed by RGCs (Cohen-Cory et al., 1996; Frost et al., 2001; Rohrer et al., 2001; Vecino et al., 2002), and that RGCs express the cognate TrkB receptor (Cohen-Cory et al., 1996; Suzuki et al., 1998; Rohrer et al., 2001; Vecino et al., 2002; Marshak et al., 2007), the clearly defined developmentally regulated change in BDNF transcript levels in different birth-dated RGC cohorts reported here is a new finding. Importantly, growing CNS-derived RGCs also exhibited increased expression of transcripts associated with survival- and growth-related pathways downstream of TrkB. These included mRNA for signaling proteins downstream of TrkB-Ras, which promote survival and neurite outgrowth through MAPK phosphorylation of CREB (Mayr and Montminy, 2001), and lead to upregulation of a wide range of anti-apoptotic genes, including BDNF itself (Korsmeyer, 1999; Mayr and Montminy, 2001). In actively growing RGCs, pathways downstream of TrkB-Akt were also activated, consistent with a role for Akt1 in inhibiting pro-apoptotic factors and promoting survival in response to neurotrophic stimulation (Korsmeyer, 1999; Brunet et al., 2001).

TrkB expression has also been reported to be developmentally regulated (Rickman and Brecha, 1995; Ugolini et al., 1995), but we were unable to detect any change. Previous studies in rat have shown that exogenous application of BDNF or the related neurotrophin NT-4/5 temporarily protects P4/P5 RGCs after central target ablation (Cui and Harvey 1995; Spalding et al., 1998), and BDNF supports the survival of cultured neonatal RGCs (this study; cf. Johnson et al., 1986; Meyer-Franke et al., 1995; Cohen-Cory et al., 1996), which it should be noted have also been axotomized during the process of isolation and cell culture. Furthermore, BDNF is expressed in the SC and is retrogradely transported by RGCs (Ma et al., 1998; von Bartheld, 1998; Frost et al., 2001; Rohrer et al., 2001; Marotte et al., 2004). Our new data are thus consistent with the proposal that BDNF has an important role in maintaining cell-autonomous RGC survival before axonal ingrowth into central targets. Such an interpretation echoes the autocrine (and/or paracrine) supply of neurotrophins that occurs in peripheral sensory neuron populations prior to target innervation (Wright et al., 1992; Davies, 1996).

Our second line of evidence comes from the *in vitro* data showing that the dependency of purified P1 RGCs on exogenous BDNF for survival are related to their date of birth and thus their axon growth status at the time of isolation. In the absence of exogenous BDNF the number of cultured E15 BrdU^+^ RGCs declined by >50% between 24 and 48 h while late-born E18 BrdU^+^ RGCs survived for at least 48 h. Additionally, E18 RGCs maintained expression of endogenous BDNF throughout their time in culture, consistent with these cells still being target independent. Thus temporal differences in the acquisition of target dependency and onset of PCD in early-born versus late-born RGCs are maintained in culture and in the absence of *in vivo* environmental signals, suggesting that such differences may be intrinsically determined (cf. Davies et al., 1987; Vogel and Davies 1991; Davies, 1996).

Despite these various observations suggesting BDNF via TrkB acts as a survival factor for developing RGCs, the number of RGCs is not reduced in BDNF knock-out mice (Cellerino et al., 1997), and RGCs survive the postnatal period in mice lacking TrkB (Rohrer et al., 2001). Depletion of TrkB using fusion proteins does increase the peak rate of RGC death in neonates but does not alter final cell number (Pollock et al., 2003). After early gene deletion there may be compensation (via NT-4/5 actions and/or cytokine expression, or signaling through other receptors) that maintains RGC viability; however, conditional Cre-mediated deletion of BDNF as neurons mature also did not affect RGC viability or myelination (Rauskolb et al., 2010). Note, however, that in these mice some BDNF (5% of control) was still measurable by ELISA in cerebral cortex, but BDNF levels were not reported for retinal tissue.

It is difficult to reconcile these various observations to our current, and previously published, findings. TrkB expressed in the absence of BDNF does not trigger neuronal death and is thus not a “dependence receptor” (Nikoletopoulou et al., 2010). However, lack of ligand-induced kinase activity in TrkB receptors may alter numerous downstream pathways that indirectly mediate RGC death. RGCs do express p75 and/or the related Troy, both members of the tumor necrosis factor receptor family that possess an intracellular death domain (Mandemakers and Barres, 2005; Ahmed et al., 2006). Perhaps the well described survival effects of either BDNF or NT-4/5 on neonatal RGCs *in vitro*, or after injury *in vivo*, are related to altered expression of p75/Troy and TrkB. Yet numerous studies have documented prosurvival effects of BDNF and NT-4/5 in the normal uninjured rodent visual system; application of BDNF to the SC increased RGC survival (Ma et al., 1998), and RGC loss was reduced after intravitreal NT-4/5 injection (Cui and Harvey, 1995). Excess “target-derived” BDNF also prevented the retraction of the normally transient ipsilateral retinotectal pathway (Isenmann et al., 1999). Coinjection of blocking antibodies to BDNF and NT-4/5 in the SC in neonatal rats significantly increased RGC death, as did injection of these same antibodies into the retina, although it is important to note that this increase was considerably less after intraretinal antibody application, which is clearly to be expected if only late-born not yet target-dependent RGCs were affected (Spalding et al., 2004). Finally, Carpenter et al. (1986) and Spalding et al. (2004) described the extensive loss of neonatal RGCs after kainic acid lesions in the SC, a technique thought to cause loss of target neurons in the SC without concomitant axonal injury.

### The switch to target dependency in developing RGCs: other growth factors

Expression of cytokine signaling-related transcripts was also significantly different between RGCs at different stages of target ingrowth. Actively growing RGCs expressed relatively higher levels of CNTFRa mRNA, a subcomponent of the cytokine receptor (Stahl and Yancopoulos, 1994), and its downstream effectors STAT1 and STAT3, known to promote survival and growth (Peterson et al., 2000; Park et al., 2004; Ng et al., 2006). There was also increased expression in transitional and growing RGCs of IL6st, another subcomponent of the cytokine receptor, and IL6R. It has been reported that exogenous CNTF does not support the survival of RGCs *in vitro* (Meyer-Franke et al., 1995) or P4/P5 RGCs *in vivo* (Spalding et al., 2005). However, intraocular application of leukemia inhibitory factor did reduce RGC death to some extent after early SC ablation (Cui and Harvey, 1995). Given the very small population of late-born RGCs, without information about birth date any positive effects of cytokines would be less obvious within the overall RGC population. It is worth noting here that in the adult rat CNTF is a powerful driver of RGC survival and long-distance axonal regeneration (Fischer, 2012; Harvey et al., 2012).

There was significantly greater expression of IGF-1R in growing versus target-dependent RGCs, and relatively greater expression of this receptor in transitional RGCs. IGF-1 is known to have protective effects on neonatal (Gutiérrez-Ospina et al., 2002) and adult (Kermer et al., 2000) RGCs, and IGF-1R expression is necessary for the expression of neurites from adult RGCs *in vitro* (Dupraz et al., 2013). Note that signaling through this receptor also involves PI3-K and Akt pathways, the latter consistently upregulated in growing RGCs (Fig. 3; Kermer et al., 2000). Transitional RGCs also appeared to express greater levels of the kinase Stk24, also known as MST3b, implicated in the regrowth of adult RGC axons (Lorber et al., 2009).

Not all transcripts for prosurvival proteins were more abundant in actively growing RGCs. Expression of genes encoding proteins downstream of TrkB via PLCg, a pathway implicated in moderating survival in response to synaptic interactions and neuronal activity (Tao et al., 1998; Ming et al., 1999; Reichardt, 2006), was reduced presumably because these actively growing RGCs had not yet formed synaptic connections. In this regard, there was a large fold difference between growing and transitional RGCs in expression of transcripts for the presynaptic protein neurexin 1 (Fig. 2*B*); this is to be expected given that this protein is needed during the process of synaptogenesis (Barker et al., 2008). Finally, we detected increased levels of pro-apoptotic Bax and GSK-3B mRNA, although the concomitantly increased levels of Akt might inhibit the function of these two proteins (Tokuoka et al., 2002; Zhou and Snider, 2006; Alabed et al., 2010). Downregulation of Akt when RGC axons reach their target could provide a rapid and sensitive mechanism for regulating RGC death.

### Interpretation of gene expression: RGCs at a transitional stage of target dependency

In comparing growing (E18/P1) to transitional (E18/P5) RGCs, many of the differences in gene expression were similar to those found for growing versus target-dependent RGCs. Significant differences were also detected between transitional and target-dependent RGCs using multivariate analysis, although such differences were fewer, with generally lower fold changes. Expression of transcripts for proteins in the PLCg pathway was reduced in target-dependent compared with transitional RGCs, including significantly lower levels of CAMK1 mRNA expression. Perhaps the effects of this activity-dependent survival pathway become more important as RGCs begin the process of target innervation.

### The development of retina-to-brain connectivity

During visual system development, BDNF also acts as a terminal arborization factor and promotes the formation and stabilization of synapses (Hu et al., 2005; Marshak et al., 2007). The high level of BDNF we observed in actively growing RGCs seems to conflict with findings that delivering BDNF to the axons of developing RGCs causes long-distance axon extension to cease and triggers collateral arborization and synapse formation (Cohen-Cory and Fraser, 1995). However, in development at least, BDNF can be delivered to the maturing retina without impeding long-distance RGC axon extension (Lom et al., 2002; Cohen-Cory et al., 2010). Perhaps the effect of BDNF on RGC axon outgrowth during development depends on whether it activates TrkB receptors in the axon terminal or the cell body (Lom et al., 2002; Cohen-Cory et al., 2010; Park and Poo, 2013). Alternatively, our new data also pointed to developmental changes in RGC sensitivity to cytokines and other factors, such as insulin-like growth factors. Such factors acting as intermediaries en route to targets could facilitate/promote axonal growth in concert with the role of BDNF in sustaining RGC viability.

Although our experiments did not determine the cause of BDNF downregulation in RGCs as they transition to dependence on target-derived trophic support, we can speculate on some of the relevant candidates identified in our expression screen. One possible candidate is the transcription factor CREB, a known driver of BDNF transcription, which is transiently switched off in RGCs in transition and may thus signal a change in transcriptional targets. In this context our initial qPCR suggested that there may be altered expression of members of the NFAT transcription factor family, important in mediating neurotrophin signaling during initial axonal growth (Graef et al., 2003). In addition numerous transcription factors and epigenetic processes including DNA methylation, histone modifications, and microRNA binding could be responsible for developmental changes in BDNF expression (Chen et al., 2003; Numakawa et al., 2010; Zheng et al., 2012; Karpova, 2014; Varendi et al., 2014). Many of these regulatory mechanisms are activity dependent and can direct the mRNA to different locations within the neuron, thus influencing protein function.

Maturational changes in post-translational processing of BDNF may also be important. ProBDNF is cleaved to mature BDNF and these different forms influence RGC axonal growth state via direct and indirect mechanisms (Cohen-Cory et al., 2010). Mature BDNF preferentially activates TrkB, directly promoting survival and axonal growth, whereas proBDNF binds to the p75NTR-sortilin receptor complex to promote apoptosis, or to the p75NTR-NgR-LINGO complex to inhibit neurite outgrowth (Mandemakers and Barres, 2005). Furthermore, the form of BDNF may indirectly change the growth state of RGCs by influencing the outcome of Eph receptor signaling as RGC axons innervate the target. ProBDNF association with p75/TROY (Marler et al., 2010) suppresses EphA-mediated arborization (Marler et al., 2010; Poopalasundaram et al., 2011), but mature BDNF signaling has the reverse effect, promoting branching via the association of the EphA receptor with TrkB (Marler et al., 2008, 2010; for review, see Cohen-Cory et al., 2010). The complex interaction of BDNF and Eph receptor signaling raises the intriguing possibility that downregulation of BDNF expression in developing RGCs may be necessary to enable retinotopic mapping in response to precisely regulated levels of target-derived BDNF (Goodhill and Richards, 1999; Feldheim et al., 2000; Marotte et al., 2004).

### Technical considerations

The experiments reported here are, to our knowledge, the first to compare mRNA expression in identified, birth-dated CNS neurons using LCM. The use of BrdU *in vivo* to help define the spatial-temporal maturation of birth-dated olfactory sensory neurons has recently been reported (Rodriguez-Gil et al., 2015), although this group did not use LCM to isolate individual neurons for gene expression profiling. As detailed in the Results, we encountered several technical issues associated with undertaking qPCR on RGCs captured from fixed retinal sections that had been processed for EdU and Brn3a. Despite the collection of over 25,000 individual RGCs to provide RNA for qPCR, RNA degradation from necessary fixation and IHC protocols likely resulted in the failed amplification and variability seen for some genes in the panel. In addition, the variance we often found between samples may reflect a number of real biological variables. Although our estimates here apply to the time required to reach the SC, RGC axons also grow into other targets including the lateral geniculate nucleus and other visual brain regions en route. This, together with delivery of EdU over several hours, may contribute to the variability observed, especially in the transitional E18/P5 cohort, even within identified RGCs of similar but not identical birthdates. Finally, as discussed above, protein function can be altered by post-transcriptional and post-translational processing, as well as by subtle changes to intracellular localization, which are not captured by our qPCR data. This may explain why some genes, such as some Bcl-2 family members, showed no clear-cut changes in expression during the process of target innervation. In future studies it will clearly be of interest to investigate protein expression and epigenetic changes in microdissected CNS neurons identified by day of neurogenesis.

## Conclusion

Single-cell laser microdissection is a powerful tool for assessing gene expression in individual cells *in situ*. To our knowledge this method has not previously been used to obtain snapshots of gene expression in developing mammalian neurons specifically identified by their day of neurogenesis, at a defined state of *in vivo* maturation. Such an approach presents technical challenges; nonetheless we have been able to show that gene expression in rat RGCs born on a particular day is correlated with their developmental stage of axonal outgrowth and target innervation. Thus studies that define developmental changes in gene expression based solely on the postconception age of the animal may not give an accurate representation of gene expression changes across development and beyond. While further work is needed to clarify and extend the present work, our data suggest that the transition to target dependency in neonatal rat RGCs is linked to the downregulation of endogenous BDNF expression and altered signaling in downstream pathways, as well as changes in responsiveness to cytokines and perhaps other growth factors. We hypothesize that the combined effects of decreased responsiveness to axogenic factors and decreased intraretinal BDNF expression allows TrkB expressing RGCs to become more sensitive to target-derived BDNF, which is important for the onset of local arbor formation and for the establishment and stabilization of ordered retinotectal connectivity.

## Synthesis

The decision was a result of the Reviewing Editor Michael Sofroniew and the peer reviewers coming together and discussing their recommendations until a consensus was reached. A fact-based synthesis statement explaining their decision and outlining what is needed to prepare a revision is listed below. The following reviewers agreed to reveal their identity: Elizabeth Coulson, Carol A. Mason

Synthesis of Reviews:

Reviewer #1

The paper eN-NWR-0044-14R1 describes an RNA analysis of retinal ganglion cells (RGCs) born at different ages and different stages of target innervation, showing there are gene transcript differences. The main conclusion, as per the authors focus, is that BDNF and genes in the TrkB pathways (albeit not exclusive to TrkB) are upregulated in neurons that are growing axons and down regulated post-contact. This is not similar to findings in SCG neurons (2008 Science Deppmann et al.,) where growth factors for competition/selection were not upregulated until after first contact.

The authors suggest that the BDNF expressed by the growing neurons keep them alive until the target is reached and then everything is down-regulated while they undergo selection. Alternatively the E15 neurons examined have developed further from target selection phase and are not becoming growth factor independent perhaps becoming reliant on a cytokine? ( as occurs with DRG neurons). Furthermore, given the connected neurons are theoretically signalling survival, it is odd that the Bcl type survival genes are not up regulated in response to the target signalling - might this be occurring just in the axon? How can these inconsistencies be explained?

As the main results is a data set, Table 1 should have some data attached to it with an indication of up or down regulation vs steady for the three time points.

Retrospectively it would have been best to look at the same population of cells E15 at the three time points as then one knows the neurons started out the same and so any transcript changes are due to development. The fact that the E18 cells are most similar to each other could be epigenetics due to their birthdate rather than they at they have not yet made final contact with the target. Nonetheless the findings are worthy of reporting and could be a nice data set for others to mine in the future, if validated. Real interpretation will require future experiments to tease out. With this in mind I think the study should provide a further overview of the results as part validation.

Overall, it is difficult to drill down to find the conclusions. The authors should try to articulate these better.

If things didn't go as predicated for known genes, can we as readers trust the changes found in other genes, so as to make hypothesis from them to test in the future? This analysis could then provide for the discussion, conclusion and abstract, additional concrete examples of what did or didn't change as predicted.

The methods are well written, and extensive. I cant really follow the dot graphs (I'm guessing one of those plots you do with huge data sets) but the bar graphs and the pathway diagrams are helpful.

Minor comment

In study 1 there was no quantification of RNA, so housekeeping genes were used, in study 2 RNA was quantified - were the house keeping genes also be assessed to validate the study 1 method

Reviewer #2

It is known that immature CNS-derived retinal ganglion cells (RGCs) become dependent on target-derived neurotrophic support as their axons reach their targets in the brain, with the tectum/superior colliculus most studied target of RGCs. The transition to target dependence has been analyzed most extensively in the PNS, and there is less information on the properties of the RGCs that change as axons grow to towards their targets. In a first period, they are independent of target-derived trophic support, then as axons near/enter their target, they transition to target dependence, finally to become dependent on the target for survival. Here the authors have labeled RGCs with BrdU or EdU at two embryonic times then sacrificed the animals at intervals corresponding to these three periods. They used laser capture micro-dissection to perform gene profiling to identify genes for factors enabling the independence and target-dependence. Although the factors identified by gene profiling are already known to be intrinsic to growing RGCs (i.e., BDNF), other novel factors of interest, both trophic and signaling factors, were pulled out in the different periods.

The authors have made amendments to the original manuscript, which I did not review. This version needs textual amendments but is otherwise will be strong contribution to the literature.

Introduction:

Lines 53-70 - Although timing of outgrowth from retina to target is cited from the fairly old literature, missing are references to target-independent features of growing RGCs and their intrinsic expression of BDNF, and later reliance on target-derived neurotrophins in the target -dependent phase (i.e., work of Cohen-Cory).

Methods:

1. lines 132-138: In the description of the laser capture experiments , "groups" is ill defined until later in this passage. Also, lines 159-173 where the numbers of cells captured is detailed, clarify from how many retinas the cells are pooled and across how many litters of pups.

2. It is unclear throughout when BrdU and EdU were used in individual experiments, whether there is a rationale for using one or the other or whether they are used strictly interchangeably.

Discussion:

1. The last two sentences in the first paragraph (lines 422-425) present a defense of the current approach. The authors should remove these sentences and simply make the introductory paragraph a positive statement on their accomplishments, as the remaining sentences do. State how their approach produced an advance over previous literature, and instead make a separate paragraph or section based on lines 422-425 and discussg the "confounding issues associated with analysis of whole retinal tissue or analysis of gene expression in vitro on cultured or cell sorted RGCs." Analysis of cell sorted RGCs is becoming increasingly common and does not have the same issues associated with whole retinal tissue analysis. Such a section could go into more detail with regard to technical caveats, namely, in analyzing gene expression in fixed and processed tissue and provide explanations of why the data resulting from this method are comparable to or better than the confounding issues alluded to with other approaches, and perhaps even propose improvements for future studies.

2. The authors should be congratulated for executing this time-consuming and technically challenging approach, but the fact that it is time consuming does not justify its relevance and importance. Thus, we suggest removing this phrase in line 425.

3. The methodology presented in the manuscript is indeed novel and impressive and provides a new way of analyzing dynamic gene expression of developing neurons relative to their birthdate and correlated axon growth stage and target dependence. This approach provides a new view of these developmental processes. A study has recently appeared using the same approach to chart the expression of odorant receptors relative to their birthdate and extent of growth along the pathway to the olfactory bulb and should be cited: Rodriguez-Gil DJ, Bartel DL, Jaspers AW, Mobley AS, Imamura F, Greer CA. Odorant receptors regulate the final glomerular coalescence of olfactory sensory neuron axons. Proc Natl Acad Sci U S A. 2015 May 5;112(18):5821-6. doi: 10.1073/pnas.1417955112. Epub 2015 Apr 20. This study did not use gene profiling to dissect differences, however.

4. The Discussion is thorough and covers the relevant literature vis a vis the trophic factors identified and previous work on the visual system, in depth. Nonetheless, given that it is ∼8.5 pages, it could be made more succinct.

Minor criticisms:

line 66: RGCs... "express" axons is odd; "project' or "extend" are less awkward.
